# Endophthalmitis Caused by α-Streptococcal Following Intravitreal Ranibizumab Biosimilar Administration Using a Prefilled Syringe

**DOI:** 10.7759/cureus.104163

**Published:** 2026-02-24

**Authors:** Kengo Utsunomiya, Yoichi Sakurada, Hiroshi Kasai, Ayane Tanaka, Kenji Kashiwagi

**Affiliations:** 1 Department of Ophthalmology, University of Yamanashi, Chūō, JPN

**Keywords:** endophthalmitis, intravitreal injection, pre-filled syringe, vascular endothelial growth factor, α-streptococcus

## Abstract

Endophthalmitis continues to represent a vision-threatening complication of intravitreal injection. Herein, we present a rare case of endophthalmitis caused by α-streptococcal following intravitreal ranibizumab biosimilar administration using a prefilled syringe. A 72-year-old woman was referred to our clinic and diagnosed with acute endophthalmitis in the left eye, following intravitreal administration of a ranibizumab biosimilar delivered via a prefilled syringe. A hypopyon was noted at a height of 3 mm, and the fundus examination of the left eye was obscured by corneal edema. The best-corrected visual acuity (BCVA) in the left eye was limited to hand motion. Urgent vitrectomy was performed with intraoperative irrigation using ceftazidime sodium (20 mg/500 mL) and vancomycin (10 mg) in the left eye. α-Streptococcal was isolated from both the aqueous humor and vitreous culture specimens. At 19 days postoperatively, the fundus was clearly visible, and the BCVA had recovered to 0.4 in the left eye, which corresponded to the pretreatment level. Urgent vitrectomy with irrigation using ceftazidime sodium and vancomycin may facilitate visual recovery in the affected eye.

## Introduction

Vascular endothelial growth factor (VEGF) comprises four isoforms, among which VEGF-125 is a key mediator in various retinal diseases [1]. Intravitreal administration of VEGF inhibitors has become the standard treatment for several retinal diseases, including neovascular age-related macular degeneration (AMD), diabetic macular edema (DME), and macular edema (ME) secondary to retinal vein occlusion [2,3]. Although more than 20 years have passed since the approval and commercial availability of the first VEGF inhibitor, pegaptanib, by the Food and Drug Administration [4], endophthalmitis remains a serious vision-threatening complication of intravitreal injection. Published studies consistently demonstrate that post‑injection endophthalmitis is a rare complication of intravitreal anti-VEGF therapy. Large multicenter analyses have reported incidence rates typically ranging from 0.01% to 0.05% per injection, confirming that the risk of infection is very low relative to the substantial therapeutic benefits of anti-VEGF agents [5].

Following the introduction of VEGF inhibitors, several strategies have been adopted to reduce the risk of endophthalmitis, such as both healthcare personnel and patient masks, pre-injection disinfection, antibiotic eye drops, adoption of prefilled syringes, and adherence to a “no-talking” protocol [5]. According to a review, the incidence of endophthalmitis associated with prefilled syringes is lower than that observed with conventional glass-vial preparations. Povidone‑iodine 5% is widely regarded as the gold‑standard antiseptic for pre‑injection preparation in intravitreal procedures. Its broad antimicrobial activity, rapid onset, and proven efficacy in reducing the risk of post‑injection endophthalmitis make it an essential component of standard aseptic technique. Numerous studies have demonstrated that proper application of povidone‑iodine to the ocular surface and eyelid margins significantly lowers the incidence of bacterial contamination during intravitreal injections. Given its strong evidence base and universal acceptance, clear documentation of povidone‑iodine use is critical when describing prophylactic measures in clinical reports [6].

Several studies have demonstrated that alpha‑hemolytic streptococci, particularly viridans group streptococci (VGS), are important causative pathogens of infectious endophthalmitis. Recent analyses have shown that VGS are increasingly associated with post‑procedural and endogenous endophthalmitis, and isolates recovered from affected eyes exhibit diverse virulence and toxin profiles [7,8].

In this report, we present a case of endophthalmitis caused by α-streptococcal following intravitreal ranibizumab biosimilar administration using a prefilled syringe.

## Case presentation

The patient, a 72-year-old woman, was referred to our clinic for a sudden vision loss and pain in the left eye due to endophthalmitis, occurring three days after intravitreal administration of a ranibizumab biosimilar agent via a prefilled syringe. She had a history of ME secondary to branch retinal vein occlusion and had received 18 intravitreal injections of VEGF inhibitors, including two doses of aflibercept 2 mg, eight doses of ranibizumab, and eight doses of ranibizumab biosimilars. She had a history of breast cancer surgery and was still under follow‑up in the breast surgery department, but she had no systemic diseases such as hypertension or diabetes.

At the initial presentation, three days after the 19th injection, the best-corrected visual acuity (BCVA) was 1.0 in the right eye and hand-motion in the left eye. Table 1 presents the anterior segment findings of both eyes at initial presentation. 

**Table 1:** Anterior and posterior findings on the initial presentation

		Right eye	Left eye
Best-corrected visual acuity (decimal format)		1.0	10.0 cm/m.m.
Intraocular pressure (mmHg)		8.6	38.1
Anterior segment	Conjunctiva	Normal	Conjunctival injection
	Cornea	Clear	Edema
	Anterior Chamber	Clear	Hypopyon 3 mm
Ocular media		Cataract	Poor visibility
Posterior pole		Normal	Obscured by media opacity

**Figure 1:**
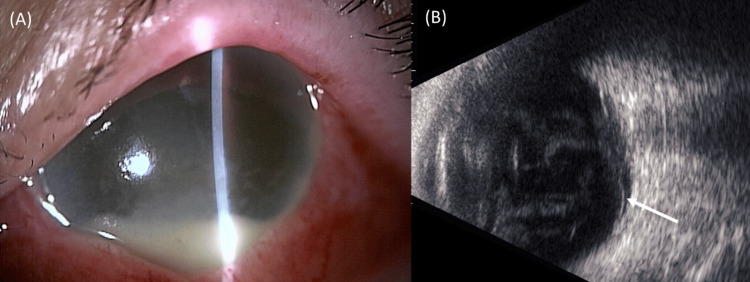
Clinical images of the left eye at the initial presentation (A) In the anterior chamber, the hypopyon is 3 mm in height. The fundus is not clearly visible due to the corneal edema. (B) Medium‑reflective echoes within the vitreous cavity, consistent with inflammatory cells, were observed. In addition, serous or tractional retinal detachment (white arrow) and retinal thickening were present in the posterior pole. Increased choroidal reflectivity was also noted in the same region on the B-mode echo.

In the left eye, fundus visualization was obscured by a dense vitreous opacity. Figure 1 illustrates images of the left eye at the initial presentation.

At the previous clinic, intravitreal injections were performed in a sterile operating room, with both the patient and physician wearing masks and caps. Injections were performed under sterile conditions. Pre‑injection antisepsis was performed using 5% povidone‑iodine, which was applied to the conjunctival sac and periocular skin. Following the procedure, antibiotic drops were administered four times daily.

**Figure 2:**
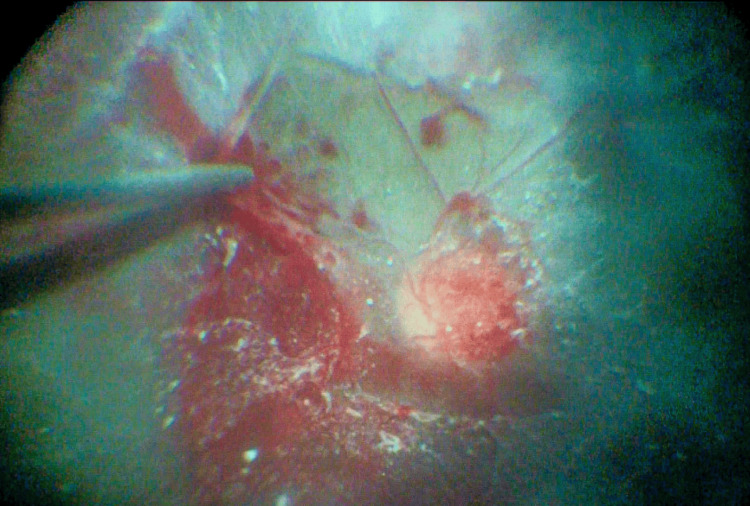
Intraoperative image of the fundus The intraoperative image reveals retinal hemorrhages beneath the posterior hyaloid visualized with triamcinolone. The vitreous cutter is used to remove the proliferative membrane carefully.

Urgent surgery, including phacoemulsification and vitrectomy, was performed on the left eye at the initial presentation. Endolaser photocoagulation and SF6 20% gas tamponade were additionally performed to treat retinal breaks caused by proliferative tissues. Figure 2 presents an intraoperative fundus image of the left eye.

During surgery, samples of aqueous humor from the anterior chamber and the vitreous humor were collected for bacterial culture and Gram staining. Ceftazidime was also administered intraoperatively. Irrigation with balanced salt solution included ceftazidime sodium (20 mg/500 mL) and vancomycin (10 mg). α-Streptococcal was isolated from both aqueous humor and vitreous culture specimens. However, bacterial culture testing did not reveal any drug susceptibility.

Postoperatively, cefazoline sodium (3 g/day) was administered by intravenous drip for three days, and topical levofloxacin (1.5%) and betamethasone (0.1%) were applied four times daily.

**Figure 3:**
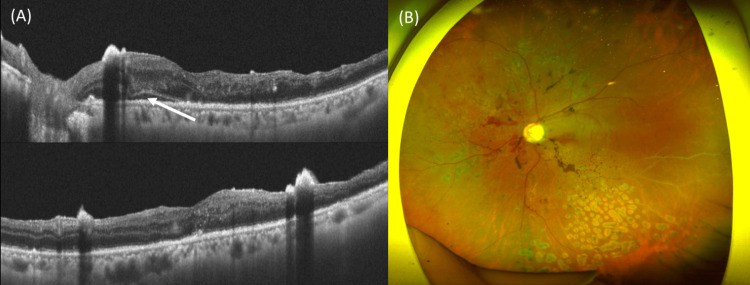
Clinical images obtained at one month postoperatively (A) Optical coherence tomography reveals subretinal fluid (a white arrow) and residual proliferative tissue corresponding to hyperreflective points. (B) Wide-field fundus photography reveals retinal hemorrhage around the optic disc and photocoagulation scars inferior to the vascular arcade.

At 19 days postoperatively, inflammation in the anterior chamber and vitreous had nearly resolved, and the BCVA in the left eye was 0.05 (decimal). At 45 days postoperatively, the inflammation had completely resolved, and the BCVA in the left eye improved to 0.4 in the decimal format. Figure 3 presents the clinical images obtained at 45 days postoperatively.

## Discussion

In cases of postoperative exogenous endophthalmitis, the most commonly isolated causative organisms are gram‑positive cocci, particularly coagulase‑negative staphylococci, which originate from the patient’s own ocular surface or periocular skin. *Staphylococcus aureus* and various *Streptococcus* species are also encountered, although less frequently. Notably, streptococcal infections occur less often than staphylococcal infections but are associated with more fulminant clinical courses and poorer visual outcomes [7-10].

In this case, endophthalmitis occurred following the use of a prefilled syringe, despite appropriate ocular surface and periocular antisepsis with 5% povidone‑iodine. Antisepsis with 5% povidone‑iodine remains the most effective and universally accepted measure for preventing exogenous endophthalmitis in ophthalmic procedures. Its broad antimicrobial activity rapidly reduces conjunctival and periocular bacterial load, outperforming all other prophylactic strategies. Clinical studies consistently demonstrate that povidone‑iodine application before intraocular surgery significantly lowers postoperative infection rates and is considered the gold standard of endophthalmitis prophylaxis. Even when compared with routine or selective antibiotic prophylaxis, povidone‑iodine disinfection provides equivalent or superior protection, underscoring its essential role in modern ophthalmic practice [10]. Theoretically, sterile medication administration using a prefilled syringe reduces the risk of contamination and lowers the incidence of endophthalmitis compared with administration from a glass vial. A review reported that the incidence of endophthalmitis was 0.017% (one in 5882) and 0.052% (one in 1923) with prefilled syringes and glass-vial preparation, respectively [5]. During the intravitreal injection, both the patients and physicians wore masks. However, a definitive conclusion has not yet been reached regarding the effect of universal masking on the risk of endophthalmitis. In general, physician mask use has been recommended, as it may reduce the transmission of nasopharyngeal and oral flora to the eye during intravitreal injections. However, patient masking may paradoxically increase the risk of endophthalmitis, as oral flora can escape from the superior edge of an improperly fitted mask. Consequently, the “no talking” protocol is recommended by the expert consensus of EURETINA [5]. In this instance, wearing a mask may have been a contributing risk factor for the development of endophthalmitis.

Recently, second-generation VEGF inhibitors, including brolucizumab, faricimab, and aflibercept (8 mg), have become standard treatments for neovascular AMD and DME [11-13]. They strongly impact the resolution of exudation; however, intraocular inflammation (IOI) is a major concern following intravitreal administration. A few reports have demonstrated IOI after first-generation VEGF inhibitors, including ranibizumab and aflibercept [14,15]. Therefore, distinguishing endophthalmitis from IOI is important. However, in this case, hypopyon and severe inflammation in the anterior chamber developed 3 days after intravitreal administration, and the fundus was not visible owing to vitreous exudates. Severe inflammation and hypopyon in the anterior chamber and vitreous humor are rarely observed in patients with IOI. The hypopyon was considered indicative of infection, prompting urgent vitrectomy with phacoemulsification and aspiration. Ultimately, the BCVA improved to 0.4 in the decimal format, approximating pre-endophthalmitis levels. Therefore, urgent surgical intervention may prevent vision loss caused by endophthalmitis.

The historical context of postoperative endophthalmitis management is also important when interpreting the present case. The Endophthalmitis Vitrectomy Study (EVS), published in 1995, remains a landmark trial that continues to influence current treatment strategies. Although surgical techniques and diagnostic tools have advanced considerably since its publication, EVS established several principles that still underpin modern practice. In particular, EVS demonstrated that the decision to perform early vitrectomy should be guided by the presenting visual acuity. Patients presenting with only light‑perception vision benefited significantly from immediate vitrectomy, whereas those with hand‑motion vision or better could be effectively managed with vitreous tap and intravitreal antibiotics alone. The study also standardized the use of intravitreal vancomycin and ceftazidime, which remain central components of empirical therapy today [16].In this case, although the affected eye presented with hand‑motion vision, retinal detachment was suspected preoperatively. Therefore, pars plana complete vitrectomy was performed with intraoperative irrigation of vancomycin and ceftazidime, resulting in an improvement in visual acuity. As recommended by the EVS, we selected ceftazidime and vancomycin as the intravitreal antibiotics.

The selection of a tamponade agent may play an important role in the management of bacterial endophthalmitis. A systematic review has reported that silicone oil exerts a bacteriostatic effect by limiting aqueous space, reducing nutrient availability, and creating an environment unfavorable for bacterial proliferation [17]. However, we did not use silicone oil as a tamponade agent in this case. This decision was made because no whitening of the major posterior pole vessels was observed intraoperatively, and the vascular whitening was limited to the peripheral retina. Therefore, we considered that the eye had a high likelihood of recovery even without the bacteriostatic effect of silicone oil. In this case, intravenous cefazolin sodium (3 g/day) was administered for three days postoperatively; however, the effectiveness of systemic antibiotics after bacterial endophthalmitis remains unclear due to the lack of strong evidence, and practices vary among countries and institutions [18]. Therefore, the extent to which this treatment contributed to the outcome is uncertain. Moreover, the efficacy of systemic antibiotics in exogenous endophthalmitis remains a matter of debate, and no clear guidelines currently exist [19].

The limitations of this case report include the fact that the management in this case did not fully conform to the recommendations of the EVS. Although empirical treatment is supported by the EVS, more recent reports have described cases in which early vitrectomy is considered a preferable therapeutic approach. While the EVS provides valuable clinical guidance, intravitreal injections have increased dramatically in recent years, and post-intravitreal injection endophthalmitis has likewise become more common. However, because intravitreal injections were not performed as frequently at the time the EVS was conducted, the EVS does not address endophthalmitis following intravitreal injections, and its recommendations cannot be automatically applied to such cases [20]. Furthermore, current evidence supports the use of broader antimicrobial options, such as intravitreal moxifloxacin, as well as adjunctive intravitreal dexamethasone, both of which may significantly improve clinical outcomes

## Conclusions

We experienced a rare case of endophthalmitis caused by α-streptococcal following intravitreal ranibizumab biosimilar administration. Although the exact cause is unknown, the patient's wearing a mask is a possible contributing factor. Owing to the urgent vitrectomy with irrigation of ceftazidime sodium and vancomycin, the BCVA recovered to the pretreatment level. When intravitreal injection of a VEGF inhibitor is performed, the "no talking protocol" without patients' mask wearing should be kept.

## References

[1] Ferrara N (2009). VEGF-A: a critical regulator of blood vessel growth. Eur Cytokine Netw.

[2] Hang A, Feldman S, Amin AP, Ochoa JA, Park SS (2023). Intravitreal anti-vascular endothelial growth factor therapies for retinal disorders. Pharmaceuticals (Basel).

[3] Cheema AA, Cheema HR (2024). Diabetic macular edema management: a review of anti-vascular endothelial growth factor (VEGF) therapies. Cureus.

[4] Gragoudas ES, Adamis AP, Cunningham ET Jr, Feinsod M, Guyer DR (2004). Pegaptanib for neovascular age-related macular degeneration. N Engl J Med.

[5] Louis AM, Ali AM, Patel SB (2023). Impact of prefilled syringes and masking on postintravitreal injection endophthalmitis. J Vitreoretin Dis.

[6] Uzzan J, Mapani A, Cox O, Bagijn M, Saffar I (2024). Clinical outcomes and experiences with prefilled syringes versus vials for intravitreal administration of anti-VEGF treatments: a systematic review. Ophthalmol Ther.

[7] Yang Y, Wong Y, Li Y (2023). Clinical features, antibiotic susceptibilities, and outcomes of endophthalmitis caused by streptococcal infection: children vs. adults. Antibiotics (Basel).

[8] Yang Y, Sui W, Duan F (2022). Post-traumatic endophthalmitis caused by streptococcus species in preschool children: clinical features, antibiotic susceptibilities and outcomes. Eye (Lond).

[9] Kuriyan AE, Weiss KD, Flynn HW Jr, Smiddy WE, Berrocal AM, Albini TA, Miller D (2014). Endophthalmitis caused by streptococcal species: clinical settings, microbiology, management, and outcomes. Am J Ophthalmol.

[10] McCannel CA (2011). Meta-analysis of endophthalmitis after intravitreal injection of anti-vascular endothelial growth factor agents: causative organisms and possible prevention strategies. Retina.

[11] Lanzetta P, Korobelnik JF, Heier JS Intravitreal aflibercept 8 mg in neovascular age-related macular degeneration (PULSAR): 48-week results from a randomised, double-masked, non-inferiority, phase 3 trial. Lancet.

[12] Dugel PU, Koh A, Ogura Y (2020). HAWK and HARRIER: phase 3, multicenter, randomized, double-masked trials of brolucizumab for neovascular age-related macular degeneration. Ophthalmology.

[13] Heier JS, Khanani AM, Quezada Ruiz C (2022). Efficacy, durability, and safety of intravitreal faricimab up to every 16 weeks for neovascular age-related macular degeneration (TENAYA and LUCERNE): two randomised, double-masked, phase 3, non-inferiority trials. Lancet.

[14] Rosenfeld PJ, Brown DM, Heier JS, Boyer DS, Kaiser PK, Chung CY, Kim RY (2006). Ranibizumab for neovascular age-related macular degeneration. N Engl J Med.

[15] Heier JS, Brown DM, Chong V (2012). Intravitreal aflibercept (VEGF trap-eye) in wet age-related macular degeneration. Ophthalmology.

[16] (1995). Results of the Endophthalmitis Vitrectomy Study. A randomized trial of immediate vitrectomy and of intravenous antibiotics for the treatment of postoperative bacterial endophthalmitis. Endophthalmitis Vitrectomy Study Group. Arch Ophthalmol.

[17] Sinisi F, Della Santina M, Loiudice P, Figus M, Casini G (2022). The role of silicone oil in the surgical management of endophthalmitis: a systematic review. J Clin Med.

[18] Surawatsatien N, Kasetsuwan P, Pruksacholavit J (2025). Systematic review of clinical practice guidelines for post-cataract surgery endophthalmitis prophylaxis from 2008-2023. Clin Ophthalmol.

[19] Grzybowski A, Turczynowska M, Schwartz SG, Relhan N, Flynn HW Jr (2020). The role of systemic antimicrobials in the treatment of endophthalmitis: a review and an international perspective. Ophthalmol Ther.

[20] Merani R, Johnson MW, McCannel CA, Flynn HW Jr, Scott IU, Hunyor AP (2022). Clinical practice update: management of infectious endophthalmitis after intravitreal anti-VEGF injection. J Vitreoretin Dis.

